# Diagnose this

**Published:** 2013

**Figure:**
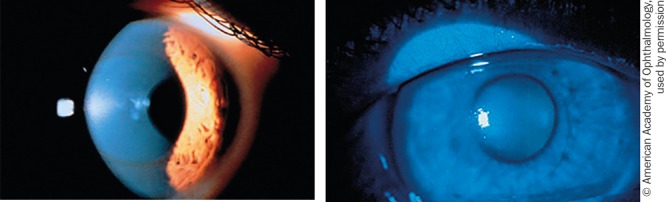


A 19-year-old college student complains of poor vision. He states that he has long been nearsighted but that his glasses have recently required several changes, and even with his most recent correction, he is having difficulty. Examination reveals a best-corrected acuity of 20/40 with spectacle correction. The results of slit lamp biomicroscopy are shown.

**What is the most likely diagnosis?**

- □ Keratoconus
- □ Terrien's marginal degeneration
- □ Pellucid marginal degeneration
- □ Keratoglobus

## ANSWER

**Keratoconus is the most likely diagnosis**.

The patient's history and corneal appearance on biomicroscopy are most consistent with keratoconus. Progressive myopia, multiple spectacle or contact lens changes, and a qualitatively unsatisfactory best-corrected acuity suggest early keratoconus. The presence of a distinct Fleischer ring representing iron deposition at the level of the basal epithelium is diagnostic of this disorder.

Pellucid marginal degeneration is a distinct disorder in the spectrum of noninflammatory ectasias of the cornea; however, it differs from keratoconus in that the thinnest area of the cornea is not at the apex of the cone, but rather in a crescentic distribution near the inferior limbus. The effect of this pathologicprogressive and marked against-the-rule astigmatism.

Generally, there is no circular iron deposition or apical reticular scarring as in keratoconus. Keratoglobus is a diffuse thinning of the ocular coats, including cornea and sclera. Patients with keratoglobus often have a markedly steepened cornea, a blue sclera, and a tendency for corneal rupture with trauma. There is also an association with collagen fragility syndromes.

Terrien's marginal degeneration is an inflammatory condition that includes peripheral vascularisation, intracorneal lipid deposition, and nonulcerative thinning and ectasia of the corneal periphery.

